# Interstitial Inorganic Phosphate as a Tumor Microenvironment Marker for Tumor Progression

**DOI:** 10.1038/srep41233

**Published:** 2017-01-24

**Authors:** Andrey A. Bobko, Timothy D. Eubank, Benoit Driesschaert, Ilirian Dhimitruka, Jason Evans, Rahman Mohammad, Elena E. Tchekneva, Mikhail M. Dikov, Valery V. Khramtsov

**Affiliations:** 1 In Vivo Multifunctional Magnetic Resonance center, Robert C. Byrd Health Sciences Center, West Virginia University, Morgantown, WV 26506, United States; 2 Department of Biochemistry, West Virginia University School of Medicine, Morgantown, WV 26506, United States; 3 Department of Microbiology, Immunology & Cell Biology, West Virginia University School of Medicine, Morgantown, WV 26506, United States; 4 Dorothy M. Davis Heart & Lung Research Institute, and Department of Internal Medicine, The Ohio State University, Columbus, OH 43210, United States; 5 Division of Medical Oncology, Department of Internal Medicine, Ohio State University Medical Center, The James Comprehensive Cancer Center, Columbus, OH 43210, United States

## Abstract

Noninvasive *in vivo* assessment of chemical tumor microenvironment (TME) parameters such as oxygen (*p*O_2_), extracellular acidosis (pH_e_), and concentration of interstitial inorganic phosphate (Pi) may provide unique insights into biological processes in solid tumors. In this work, we employ a recently developed multifunctional trityl paramagnetic probe and electron paramagnetic resonance (EPR) technique for *in vivo* concurrent assessment of these TME parameters in various mouse models of cancer. While the data support the existence of hypoxic and acidic regions in TME, the most dramatic differences, about 2-fold higher concentrations in tumors vs. normal tissues, were observed for interstitial Pi - the only parameter that also allowed for discrimination between non-metastatic and highly metastatic tumors. Correlation analysis between [Pi], *p*O_2_, pH_e_ and tumor volumes reveal an association of high [Pi] with changes in tumor metabolism and supports different mechanisms of protons and Pi accumulation in TME. Our data identifies interstitial inorganic phosphate as a new TME marker for tumor progression. Pi association with tumor metabolism, buffer-mediated proton transport, and a requirement of high phosphorus content for the rapid growth in the “growth rate hypothesis” may underline its potential role in tumorigenesis and tumor progression.

In recent decades, cancer research has experienced a paradigm shift from focusing exclusively on a seemingly obvious target, malignant cells, toward appreciation of key roles of the tumor microenvironment (TME) in cancer progression and therapy^1^. TME includes the physical components such as extracellular matrix, blood vessel endothelium, stromal and inflammatory cells, other supporting structures of the particular organ, and physiological components such as oxygen, pH, nutrients, waste products, signaling molecules, growth and pro-tumorigenic factors. Among important physiological parameters of the TME, tissue hypoxia^2^ and acidosis^3^,^4^ are well recognized hallmarks in solid tumors associated with changes in metabolic pathways; including a higher dependence on glycolysis^5^. The glycolytic phenotype of cancer cells that utilize glycolysis and lactic fermentation despite the presence of appropriate oxygen supply are features shared by nearly all tumors. Recently, metabolic alterations in cancer and, in particular the role of aerobic glycolysis first described by Otto Warburg in the 1920’s, are receiving increasing attention. The dominant perception that ATP generated through anaerobic glycolysis is needed for a high level of oncogene-induced proliferation is not unequivocally supported by available data. Alternatively, it has been proposed that the glycolytic pathway provides necessary equivalents of carbon and electrons from NADPH formed via interconnecting the pentose phosphate pathway for the synthesis of biomass. One of the consequences of a metabolic shift towards upregulated glycolysis and NADPH formation is a high concentration of glutathione, a major intracellular redox buffer.

High reducing capacity, hypoxia and acidosis of extracellular TME expose both cancer and non-transformed cells to elevated production of reactive oxygen species while providing cancer cells with a selective advantage^6^. Specifically, TME acidosis has been proposed to be selectively more toxic to normal parenchymal tissues compared with invading cancers^7^. Noninvasive *in vivo* assessment of TME parameters such as oxygen (*p*O_2_) and extracellular acidosis (pH_e_) may provide unique insights into biological processes in tumors, tumor progression, aggressiveness and efficacy of anticancer therapies. In particular, concurrent multiparameter measurements allow for the analysis of correlation between the TME parameters^8^. In this work, we employ an electron paramagnetic resonance (EPR) technique for *in vivo* concurrent assessment of *p*O_2_, extracellular pH (pH_e_) and concentration of interstitial inorganic phosphate (Pi) in TME in animal models of cancer. This technique is based on application of a recently developed soluble multifunctional paramagnetic probe providing unsurpassed opportunity for *in vivo* multiparameter measurements^9^,^10^ (see Fig. 1). Our data identifies interstitial inorganic phosphate as a new marker of tumor progression. The associate of [Pi] with tumor metabolism^11^,^12^, buffer-mediated proton transport^13^, and a requirement of high phosphorus content for the rapid growth in the “growth rate hypothesis^14^” may underline its potential role in tumorigenesis, tumor progression and metastasis.

## Results

### *In vivo* EPR profiling of tissue microenvironment in normal mammary glands

Concurrent multiparameter measurements in the tissue microenvironment of normal mammary gland performed in FVB/N wild type mammary glands (n = 23) yield the mean values being equal to 58 ± 3 (SE) mmHg of *p*O_2_, pH_e_ = 7.1 ± 0.03 (SE) and [Pi] = 0.84 ± 0.07 mM (SE) as shown in Fig. 2. Individual measurements show significant biological variations of these parameters around the mean values. Simultaneous data acquisition of all three parameters using the same probe allows for correlation analysis between these parameters as presented in Fig. 3. A positive correlation is found between *p*O_2_ and pH_e_ in agreement with a higher contribution of glycolysis in tissue energy metabolism at lower *p*O_2_ resulting in interstitial lactic acidosis. In addition, a negative correlation between *p*O_2_ and [Pi] may also reflect changes in bioenergetic status of the tissue. A decrease of [NTP]/[Pi] ratio accompanied with [Pi] increase has been previously used as an indirect measure of the tissue bioenergetic status assessed with ^31^P NMR^12^. No significant correlation has been observed between interstitial pH_e_ and [Pi] values. The observed correlations between *p*O_2_ and both pH_e_ and [Pi] have been further supported by establishing anoxic conditions in interstitial fluids by intratissue (i.t.) injection of an oxygen-consuming enzymatic system of glucose/glucose oxidase (Fig. 3, red symbols).

### EPR profiling of TME in a mouse model of spontaneous breast cancer

We performed multiparameter measurements in the TME of MMTV-PyMT transgenic mice which spontaneously develop breast cancer and emulate human tumor staging^15^. The mean values of *p*O_2_ = 50 ± 3 mmHg (SE) and pH_e_ = 6.99 ± 0.03 (SE) are slightly lower compared to corresponding values in normal mammary glands supporting an appearance of hypoxic and acidic regions in the tumor (see Fig. 2). The most dramatic changes were observed for concentration of interstitial Pi measured in TME, mean value [Pi] = 1.8 ± 0.2 mM (SE), compared to that in normal mammary gland, [Pi] = 0.84 ± 0.07 mM (SE). The individual measurements show significant variations around the mean values which allows correlation analysis between these three parameters and tumor volume as presented in Figs 4 and 5. No significant correlation has been observed between tumor volume and any of the measured TME parameters (Fig. 4).

In contrast to normal mammary glands, no significant correlation was found between *p*O_2_ and pH_e_ in tumors (Fig. 5a), which might be attributed to tumor reliance on glycolysis independent of oxygen concentration. In turn, negative correlation between *p*O_2_ and [Pi] (Fig. 5b) may reflect changes in bioenergetic status of the tissue related to changes in oxygen supply. No significant correlation has been observed between interstitial pH_e_ and [Pi] values (Fig. 5c). The observed correlation between *p*O_2_ and [Pi] has been further supported by establishing anoxic conditions in interstitial space by intratissue (i.t.) injection of oxygen-consuming enzymatic system of glucose/glucose oxidase (Fig. 5c, red symbols).

### EPR profiling of TME in non-metastatic and metastatic tumor xenografts

We compared subcutaneous (s.c.) xenografts established in immune deficient mice by injecting human lung adenocarcinoma cells that differ in their metastatic properties: while it is extremely rare for parental PC14 cells to develop metastases, PC14HM cells derived from distant metastases are highly metastatic to various organs including lung^16^. While PC14 cells form well encapsulated s.c. tumors, PC14HM tumors demonstrate high invasiveness as seen by the invasive boarder (Fig. 6b). PC14HM cells also develop a significantly higher number of lung tumor nodules upon intravenous injection (Fig. 6c). Both tumor xenografts grow at similar rates (Fig. 6a) and do not differ in the degree of vascularization (Fig. 6e) allowing for comparison of their TME parameters dependent on tumor volume.

Figure 7 shows the dependence of individual measurements of TME *p*O_2_, pH_e_ and [Pi] on tumor volume. In contrast to spontaneous developing tumors, both PC14 and PC14HM xenografts showed a correlation between each *p*O_2_ and [Pi] with tumor volume. This might be due to a modification of the normal tissue microenvironment during tumor progression after injection of malignant cells. The measured values of pO_2_ and pH_e_ are not significantly different between the PC14 and PC14HM xenografts. Extracellular inorganic phosphate concentration, [Pi], was the only discriminating parameter between non-metastatic and metastatic tumors, being higher in the highly metastatic PC14HM xenografts (Fig. 7c).

Correlation between *p*O_2_, pH_e_ and [Pi] are shown in Fig. 8. Similar to spontaneous developing tumors, the only significant correlation was found between *p*O_2_ and [Pi] (Fig. 8b).

## Discussion

Noninvasive *in vivo* assessment of chemical TME parameters such as oxygen, *p*O_2_, extracellular acidosis, pH_e_, and concentration of interstitial inorganic phosphate, Pi, may provide unique insight into biological processes in solid tumors. However, until now there were no available techniques for the concurrent assessment of these physiologically important chemical TME parameters in living subjects. Recently, we designed a trityl paramagnetic probe^9^,^10^ with unique spectral properties that allows for simultaneous monitoring of tissue pO_2_, pH_e_ and [Pi]. In this work, this probe was successfully used *in vivo* in animal models of cancer using low-field L-band EPR spectroscopy that allowed for a few cm tissue depth penetration of microwave field.

Comparison of the mean values of pO_2_ and pH_e_ in TME of MMTV-PyMT transgenic mice which develop spontaneous breast cancer with the corresponding parameters in normal mammary glands of their PyMT(-) normal age-matched littermates shows slightly lower values in tumors supporting the existence of hypoxic and acidic regions in TME in agreement with numerous literature data^10^,^17^,^18^,^19^. However, the most dramatic difference, about 2-fold higher concentration in tumors compared to normal mammary glands, was observed for interstitial Pi. When we used the same approach in tumor xenograft models, the only parameter which allowed for discrimination between non-metastatic PC14 and highly metastatic PC14HM tumors was interstitial Pi, the concentration of which was significantly higher in the metastatic tumors. The observed elevated Pi concentration in TME of breast tumors vs. normal mammary glands, and in highly metastatic tumor xenografts compared to non-metastatic tumors raises questions about the role of interstitial inorganic phosphate in tumor progression and metastasis. Among the other factors, alterations in interstitial fluid pressure and/or tumor blood flow could influence the amount of inorganic phosphate observed in the extracellular compartment^20^. We did not observe a difference in the degree of vascularization of the non-metastatic PC14 and metastatic PC14HM tumor xenografts which suggests that this factor may not contribute to the difference in [Pi] between two types of xenograft tumors (Fig. 6e,f).

Historically, endogenous phosphorus-containing metabolites related to energy metabolism (e.g. nucleoside triphosphates, NTPs, phosphocreatine and Pi) were prime candidates to be used as ^31^P magnetic resonance spectroscopy (MRS)-measured markers to help differentiate tumors from normal tissues and optimize and monitor ongoing therapies^21^,^22^,^23^. In particular, there was great interest from radiotherapists to use the above ^31^P MRS parameters as indicators of tumor tissue oxygenation. It has been found that one of the characteristic features of ^31^P MRS spectra in tumors of both animals and patients is a low [NTP]/[Pi] ratio. It was realized that ATP levels probably changed only under severe hypoxia while [Pi] might be more responsive to changes in tumor oxygenation throughout the physiological range. Note that it was concluded that dead cells contribute nothing to ^31^P NMR spectra of Pi, which is apparently not present in necrotic regions^24^. However, in these works there were no special efforts to discriminate NMR signals measured from phosphates localized in intracellular, extracellular and vascular fluids which makes the analysis of the Pi spectra less conclusive. In general, the relationships of [ATP]/[Pi] ratio to tumor tissue oxygenation was found to be obscure and less clear than in normal tissues, therefore, the initial enthusiasm for ^31^P NMR use as a tool to monitor tumor tissue oxygenation and related treatment outcome has been significantly suppressed. In subsequent studies, ^31^P NMR of endogenous phosphate became a widely-used technique to assess intracellular pH (pH_i_) *in vivo* and was the primary method to demonstrate that malignant cells keep their cytosolic pH rather slightly alkaline or neutral but not acidic^24^. Intracellular pH measurements using a ^31^P NMR signal is based on the high contribution of the signal from intracellular phosphate (e.g., by estimate in ref. 24 at least 65% of the Pi signal used for pH measurements in rat tumors comes from the intracellular fluid). If intracellular pH is significantly different from pH_e_ then the Pi peak could be broadened or even split allowing for discrimination of intracellular and extracellular Pi signals using high-filed magnetic resonance spectroscopy^20^.

An advantage of our EPR approach of Pi detection compared to ^31^P NMR is precise assignment of the measured [Pi] values to the known probe localization in interstitial extracellular space. Another important advantage of a multifunctional probe is that all parameters are measured using a single probe, therefore allowing for their correlation analyses independent of probe distribution and time of the measurements. Note that some heterogeneity in probe distribution (e.g. poor probe delivery in hypoxic/anoxic areas) may affect the average values of the parameters measured in the spectroscopic modality and, therefore, partially mask the potential differences between the parameters or decrease significance in correlation analysis. Nevertheless, we found that interstitial [Pi] negatively correlates with oxygen partial pressure, *p*O_2_, in all tissues: normal mammary gland (Fig. 3b), spontaneous breast tumors in MMTV-PyMT mice (Fig. 5b), and PC14 and PC14HM tumor xenografts (Fig. 8b). Establishing anoxic conditions in interstitial space by intratissue injection of oxygen-consuming enzymatic system of glucose/glucose oxidase further increased Pi levels (Figs 3b and 5b, red symbols). These observations are in agreement with association of high Pi content (and low [NTP]/[Pi] ratio) with changes in bioenergetics status upon lower oxygen supply^12^,^21^.

Interestingly, a positive correlation was observed between tissue *p*O_2_ and pH_e_ in the normal mammary glands (Fig. 3a) apparently reflecting higher contribution of glycolysis in tissue energy metabolism at lower *p*O_2_ accompanied by interstitial lactic acidosis. However, in contrast to normal mammary glands, no significant correlation was found between *p*O_2_ and pH_e_ in both spontaneous tumors (Fig. 5a) and tumor xenografts (Fig. 8a). In our opinion, this is a direct *in vivo* experimental demonstration of the Warburg effect - tumor reliance on glycolysis independent of oxygen concentration.

It is noteworthy that in all tumor tissues and normal mammary glands, we did not observe a correlation between pH_e_ and [Pi]. Apparently, this suggests that while both interstitial proton and phosphate concentrations are affected by metabolic alterations, the specific pathways of their accumulation in tissue microenvironment can be different.

Tumor acidosis is often considered to be a consequence of significant tumor metabolic reliance on glycolysis, in part resulting in higher lactic acid production in tumors. Nevertheless, simple quantitative analysis shows that a substantial quantity of acid equivalents production in tumor tissue comes from carbonic acid produced via oxidative metabolism^13^. An alternative explanation for the acidity of TME is an insufficient rate of proton transport from the site of generation, cancer cells, to capillaries due to a low density of tumor blood vessels. While a carrier (buffer)-mediated H^+^ diffusion has been proposed to play a major role in proton transport in the TME^13^,^25^, the contribution of interstitial phosphate buffer in proton transfer might be significantly larger, possibly up to the order of magnitude compared to current estimates, for the following reasons. First, according to our measurements, Pi concentrations in TME can be up to several fold higher compared to normal tissues. Second, TME acidosis increases the concentration of the protonated H_2_PO_4_^−^ form of phosphate, a proton carrier, by an additional factor of 2–3 (for ΔpH ≈ 0.3–04).

The absence of correlation between pH_e_ and [Pi] may be not surprising. While Pi can be considered as a major proton carrier facilitating proton transfer from malignant cells towards capillaries, bicarbonate buffer has a much higher concentration (10–30 mM^26^) compared to phosphate in interstitial fluids and, therefore, should play a primary role in pH buffering. On the other hand, concentration of carbonic acid, the protonated form of bicarbonate and proton carrier, is negligible (pKa (H_2_CO_3_) = 3.6) even at acidic tumor pH_e_, therefore bicarbonate does not contribute to proton transfer in TME. There is also an intriguing possibility discussed in the literature that acidic pH_e_ is under homeostatic control^7^,^13^.

Finally, elevated Pi levels in TME may reflect a high phosphorus content requirement for the malignant cells derived from the “growth rate hypothesis” (GRH) application to cancer^14^,^27^,^28^. Inorganic phosphate is a vital component of nucleotides, membrane phospholipids, and phosphorylated intermediates in cellular signalling. The GRH states that the carbon:nitrogen:phosphorus stoichiometry of living things, termed “biological stoichiometry^29^”, is associated with growth rate because of the elevated demands for phosphorus (P)-rich ribosomal RNA for fast growing organisms (relatively low C:P and N:P cell content). The GRH hypothesis being applied to cancer predicts that tumors are richer in phosphorus than the surrounding tissue due to the requirement of a high amount of ribosomes and other P-rich RNA components that are necessary to manufacture proteins in rapidly proliferating cancer cells. This was also suggested experimentally by significantly higher intracellular concentration of phosphorus in some types of tumors compared to somatic tissues^14^. The selective uptake of phosphorus ^32^P by tumors upon intravenous injection of radioactive phosphate has been known since the 1940 report by Marshak *et al*.^30^ supported by further observations^31^,^32^,^33^. Recently, Papaloucas *et al*.^34^ reported significantly higher P content in the serum of a group of cancer patients measured before the commencement of any treatment (12 head and neck, 13 cervical and 25 non-small cell lung cancer cases) compared to a group of 50 healthy individuals. Unfortunately, there is a current uncertainty in whether the increased P content in the serum observed in these cancer patients^34^ and elevated interstitial Pi content observed in various mouse model tumors in our work is causal.

The observed negative correlation of interstitial [Pi] and *p*O_2_ in TME (Figs 5b and 8b) show that tumor metabolism might be at least partially responsible for elevated [Pi] values. Increases in [Pi] can be a direct consequence of ATP hydrolysis upon low oxygen supply, and it may contribute to regulation of oxidative phosphorylation and glycolysis in cells^12^ and phosphate-mediated proton diffusion from cells to capillaries^13^. Nevertheless, similar negative correlations between [Pi] and *p*O_2_ in normal tissue (Fig. 3b) assumes that the other factors may also play role in establishing high interstitial Pi concentrations in TME. In this respect, note the absence of correlation of [Pi] as well as pH_e_ and *p*O_2_ with the tumor volume in TME of breast cancer in PyMT transgenic mice (Fig. 4) which may reflect the establishment of TME favorable for tumor growth at the early phase of progression of spontaneous tumors. Numerical computations in accordance to GRH suggest that indeed increased inflow of extracellular phosphorus promotes a shift towards a more rapidly proliferating cell type^28^. In contrast to the spontaneous tumors, we observed a positive correlation between [Pi] and tumor volume for tumor xenografts which might be related to the process of modifying normal tissue microenvironment during tumor progression after injection of malignant cells. Recently, Carvalho and Caramujo proposed that metastases migration may be P-driven: tumor metastasis would occur as an adaptation of tumor cells to fulfil their phosphorus requirements for rapid growth^35^.

Future studies are warranted to evaluate whether higher interstitial [Pi] in tumors compared to normal tissue, and the amplification of interstitial Pi in highly-metastatic tumors compared to non-metastatic that we documented here have physiological significance and may provide additional avenues for therapy. Existing clinical data suggest that the requirement of rapidly growing tumors for high P can have body-wide physiological consequences. Thus, oncological hypophosphatemia has been proposed to reflect transfer of serum Pi into proliferating malignant cells^36^. The authors^34^ consider that increased amount of phosphorus in the blood, when excluding other causes justifying such an increase, may indicate the existence of unidentified cancerous lesions, therefore providing a quick and inexpensive method for detection of an emergence of cancer in the body. In our opinion, measurement of interstitial [Pi] might be more sensitive and specific to cancer compared to blood measurements allowing for localized [Pi] measurements in tumor tissue. Interstitial Pi has been recently recognized as an emerging signaling molecule playing a role in promoting cell transformation and tumorigenesis via specific molecular mechanisms, including its mitogenic role that stimulates proliferation and matrix regulation, increases in ATP production through oxidative phosphorylation, and promotion of angiogenesis^37^,^38^,^39^,^40^,^41^,^42^. The observation of Pi-dependent mammographic microcalcifications for nonpalpable, early stage breast cancer^43^ also allows us to suppose that interstitial Pi might be used as a prognostic factor in tumorigenesis. Identification of interstitial Pi as a prognostic factor is of particular importance due to the current lack of predictive microscopic molecular biomarkers for evaluation of the relative risk of malignant transformation of pre-cancer lesions. Better understanding of the role of interstitial Pi in tumor progression may also help in the design of TME-targeted therapies aimed at reversing a favorable environment that tumors create for themselves, such as neutralizing tumor acidosis^44^ and controlling phosphorus and carbon availability.

## Methods

### Chemicals

Monophosphonated pTAM probe was synthesized as described in ref. 10. Glucose, glucose oxidase and salts were purchased from Sigma-Aldrich.

### L-Band electron paramagnetic resonance studies

**Figure 1:**
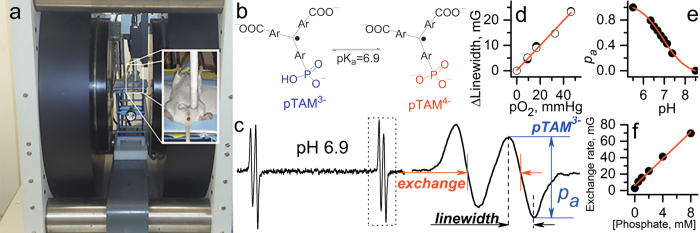
Multifunctional assessment of chemical microenvironment using trityl probes. (**a**) Set-up for *in vivo* L-band EPR measurements of the tissue microenvironment parameters in living mice. Photograph shows the anesthetized mouse between the magnets of the EPR spectrometer with the insert on the right showing placement and positioning of the loop resonator on top of the measured tissue. (**b**) The scheme of pH-dependent equilibrium between two ionization states of monophosphonated trityl probe, pTAM. (**c**) EPR spectrum of pTAM with the insert on the right showing the extended view of the high-field component. (**d**) The EPR linewidth of the pTAM is a *p*O_2_ marker (accuracy, ≈ 1 mmHg; *p*O_2_ range, 1–100 mmHg). (**e**) The fraction of protonated pTAM is a pH marker in the range from 6 to 8.0 (accuracy, ±0.05). (**f**) Dependence of proton exchange rate (expressed in mG) of the pTAM with inorganic phosphate on Pi concentration extracted by spectra simulation (accuracy, ±0.1 mM, range, 0.1–20 mM)^9^,^10^. Note interstitial extracellular localization of the pTAM probe: it does not penetrate into the cells due to bulky hydrophilic structures and the pTAM signal from the blood is not detected by EPR due to signal broadening by pTAM complexation with plasma albumin^47^.

**Figure 2:**
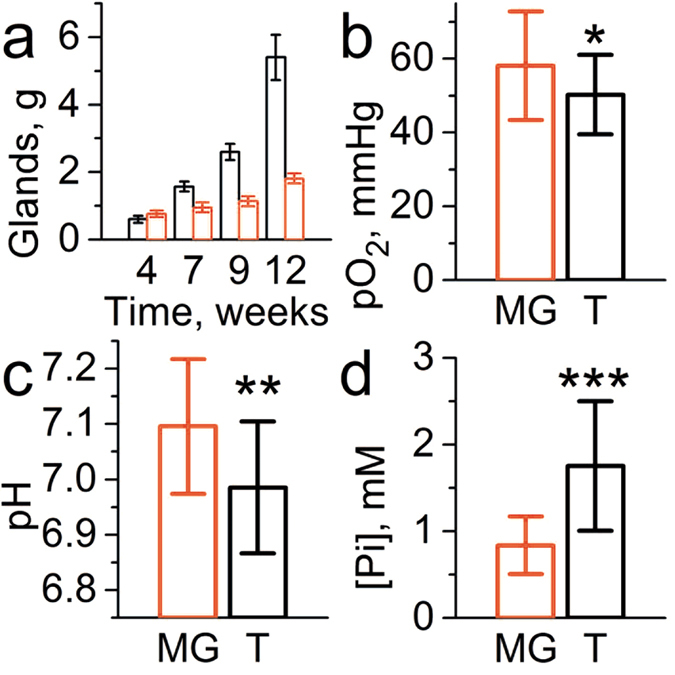
(**a**) Representative tumor burden (total weight, black columns) of MMTV-PyMT(+) mice (n = 10) and mammary glands (red columns) of their wild type PyMT(−) age-matched littermates (n = 10). The values of *p*O_2_ (**b**), pH_e_ (**c**) and [Pi] (**d**) measured in tissue environment of normal mammary gland (MG, n = 23) and tumor TME (T, n = 18). Error bars are SD. *p = 0.065, **p = 0.006, ***p = 6 × 10^−6^.

EPR measurements were performed using L-band EPR spectrometer (Magnettech, Germany). The typical instrument settings were as follows: attenuation 24 dB; modulation amplitude, 37.5 mG; modulation frequency, 100 kHz; sweep width, 0.9 G; sweep time, 20–60 s. The high-field component of pTAM EPR spectra (see Fig. 1c) was acquired during a 5–10 minute time frame.

### pH, oxygen and inorganic phosphate calibration of pTAM probe

Calibration procedure was performed similar to those published in refs 9,10 at physiological temperature (37 °C), solution ionic strength (NaCl, 150 mM), and pTAM probe concentration, 0.2 mM.

#### pH calibration

pTAM solutions were titrated by addition of a small volume of NaOH or HCl with the final dilution of sample less than 1%. pH was controlled by electrode calibrated at 37 °C using pH values for reference solution recommended by National Bureau of Standards (U.S.). Temperature of reference and titrated solutions during pH measurements was controlled using a jacketed reaction beaker attached to Lauda Circulator E100. Anoxic conditions were maintained using 10 mM addition of glucose and 100 U/mL glucose oxidase (Sigma, USA) to probe solutions.

#### [Pi] calibration

Solution of pTAM radical with pH value near *p*Ka was titrated with various concentration of phosphate. Temperature and anoxic conditions were maintained as described above.

#### pO_2_ calibration

Gas composition was controlled by bubbling solution with gas mixture delivered from gas controller (Noxygen, Germany). Solution temperature was controlled using water bath attached to the thermostat.

### General description of analysis of the EPR spectra and calculation of probe spectral parameters

Comprehensive analysis of pTAM probe EPR spectra was described in ref. 9 which takes into account the contribution of exchange processes with oxygen, protons (pH) and phosphate into the lineshape (see Fig. 1c). This allows for more accurate extraction of these parameters compared with the previously used procedure^10^. Simulated high-field components of the pTAM probe EPR spectra were least-square fitted to experimental spectra with known pH, *p*O_2_ and [Pi] values and spectral parameters were extracted and used in further calculation of unknown pH, *p*O_2_, and [Pi] values from *in vivo* experimental EPR spectra.

### Simulation of high-field EPR spectra of pTAM probe

EPR spectrum of pTAM probe is characterized by a doublet for pure pTAM^3−^ (pH ≪ pKa, see Fig. 1b for the structure) or pTAM^4−^ ionization states (pH ≫ pKa) or by quartet at pH~pKa when both states are present (see Fig. 1c). In *in vivo* studies the acquisition of full EPR spectra of pTAM probe is unpractical because of additional time used to acquire the gap between low-field and high-field components of EPR spectrum and low-resolution of full spectrum compare to one-component spectrum. Insert in Fig. 1c shows the high-filed component of the EPR spectrum used in our work for further analysis. Below we describe the EPR signal simulation procedure and calibration parameters for this specific component. Spectra were simulated using theory of exchange between several sites in non-coupled or loosely coupled systems adapted from ref. 45 as previously described for the pTAM spectra analysis^9^. Namely, EPR high-field component can be described as convolution of exchange function between high-filed component of acidic and basic ionization states of pTAM probe *V(B)* with Gaussian function of the unresolved super hyperfine structure of pTAM probe:


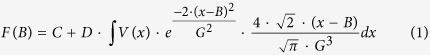


where *C* and *D* are numerical coefficients; *G* is linewidth (in Gauss) of Gaussian distribution.

The exchange function *V(B)* can be described as follows:


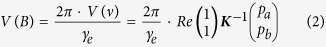



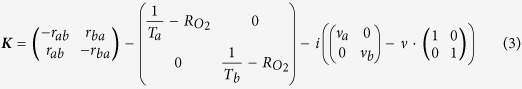


where γ_e_ = 2.8 MHz/G - electron gyromagnetic ratio; *p*_*a*_ and *p*_*b*_ *=* *(1* − *p*_*a*_) – fraction of pTAM^3−^ and pTAM^4−^ forms of pTAM probe, correspondingly; *ν*_*a*_ and *ν*_*b*_ are the frequency positions of high-field components of acidic (pTAM^3−^) and basic (pTAM^4−^) forms of pTAM EPR spectrum, namely, *ν*_*a*_ = *ν*_*0*_ − 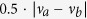 and *ν*_*b*_ = *ν*_*0*_ + 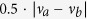, where 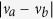 = *a*_*P*_(*pTAM*^*3−*^) − *a*_*P*_(*pTAM*^*4*−^)*, a*_*P*_(*pTAM*^*3−*^) and *a*_*P*_(*pTAM*^*4−*^) are phosphorus hyperfine splitting constant for corresponding ionization state of pTAM probe (in Hz) and ν_0_ is frequency offset; *Ta* and *Tb* are transverse relaxation times of pTAM^3−^ and pTAM^4−^ forms, correspondingly; 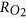 is a the rate of oxygen-induced relaxation for pTAM. Frequency exchange matrix is represented by symbols *r*_*ab*_ which are the rate of frequency exchange between spectral line *a* (acidic form of pTAM, pTAM^3−^) and b (basic form of pTAM, pTAM^4−^).

Frequency exchange between these two lines is determined by concentration of inorganic phosphate and can be described by following equation


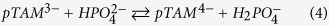


The rate of proton loss by pTAM^3−^ due to the proton transfer with reaction rate constant *k*_*f*_ equals to


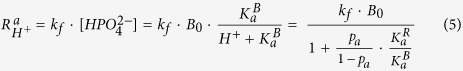


where *B*_*o*_ is a total concentration of phosphate buffer, *K*_*a*_^*B*^ and *K*_*a*_^*R*^ are ionization constants for inorganic phosphate and phosphono group of pTAM, correspondingly. Therefore the rate constant *k*_*f*_ can be found by linear approximation of dependence of 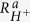 on buffer concentration, B_0_, measured at known pH value (values of *p*_*a*_, 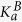, 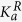 can be calculated if pH value is known). The value of reverse rate constant *k*_*r*_ of reverse reaction of pTAM^4−^ protonation can be directly calculated using following equation:


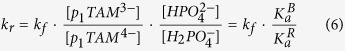


that provides simple relationship, *k*_*r*_=1.55·*k*_*f*_, for aqueous solutions with ionic strength 0.15 (150 mM NaCl).

### Fitting of simulated spectra to experimental spectra

In general case the spectrum shape *F(B)* is determined by all the discussed parameters: *C, D, p*_*a*_, *ν*_*0*_, 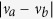, *T*_*a*_, *T*_*b*_, *G*, 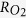, and 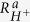. In order to decrease a number of variables, the intrinsic spectral parameters 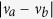, *T*_*a*_, *T*_*b*_, and *G* were first determined by least-squares fitting of the function *F(B)* to experimental EPR spectra in anoxic solutions and low buffer (Na-phosphate) concentration, i.e. 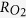 and 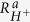 ≪ *1*/*T*_*a*_*, 1*/*T*_*b*_. The fitting of EPR spectra of anoxic 150 mM NaCl solution of 200 μM pTAM by eq. (1) yields the values a_P_(pTAM^3−^) = 3.63 G, 1/T_a_ = 23.6 mG for the spectrum acquired at pH 5.5; and a_P_(pTAM^4−^) = 3.37 G, 1/T_b_ = 9 mG and *G* = 45 mG for the spectrum acquired at pH 9.0.

Using obtained intrinsic parameters for both acidic and basic forms of pTAM (linewidth, 1/T_a_ and 1/T_b_; hyperfine splitting, *a*_*p*_), spectra of pTAM probe at different pH values were fitted to yield *p*_*a*_ values and to plot the dependence of *p*_*a*_ value on pH. Fitting of this dependence with standard titration curve yields the value of the dissociation constant, p*K*_*a*_^*R*^ = 6.9.

To assess the sensitivity of pTAM probe to oxygen partial pressure, EPR spectra were fitted with equation (1) using the intrinsic spectral EPR parameters listed above. The determined relaxation rates 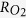 show linear dependence on pO_2_ values both for acidic (pH 5.5, pTAM^3−^) and basic (pH 9, pTAM^4−^) ionization states with the similar values of spectral oxygen sensitivity, 0.49 mG/mmHg.

Fitting the EPR spectra of pTAM at different concentrations of Na-phosphate buffer and fixed pH value close to p*K*_*a*_^*R*^ (pH 6.9) was performed and the linear approximation of 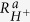 dependence on phosphate concentration was obtained yielding the value of *k*_*f*_ = 8 mG/mM.

Simulation and least-square fitting of EPR spectra were performed using Matlab software.

### Animals

All animal work was performed in accordance with the OSU IACUC approved protocol.

#### PyMT spontaneous tumor model

Twelve week-old female Friend virus B-type susceptibility/NIH (FVB/N) polyoma middle-T antigen (PyMT+/−) mice (7 animals) with spontaneously formed mammary tumors were used for *in vivo* EPR studies. For comparison of tissue microenvironments of normal mammary glands and tumors, age-matched littermate females (3 animals) absent of the PyMT oncogene (PyMT−/−) were used^15^. Mice were subject to L-band EPR spectroscopy one time per week for total of 4 weeks. Two to three mammary tumors (from PyMT(+) mice) or non-tumor bearing mammary glands (from PyMT(−) mice) were measured at one time.

#### PC14/PC14HM tumor xenograft model

We compared s.c. xenografts established in NSG (NOD scid gamma) immune deficient mice (NOD.Cg-*Prkdc*^*scid*^*Il2rg*^*tm1Wjl*^/SzJ, Jackson Laboratory, Stock No: 005557) by injecting human lung adenocarcinoma cells that differ in their metastatic properties: whereas parental PC14 cells rarely forms metastases, PC14HM cells derived from these rare lung metastases is highly metastatic to various organs including lung^16^. To evaluate the degree of tumor vascularization, tumor tissue sections were stained with peroxidase-labeled antibody to the endothelial cell marker CD34 (Santa Cruz Biotechnology, Santa Cruz, CA) and the number of tumor blood vessels was counted. Images were processed using ImageJ (National Institutes of Health, Bethesda, MD) software. Ten fields on two nonadjacent sections were counted.

*PC14 and PC14HM cells* were generously provided by Dr. Shimizu K., Department of Thoracic and Visceral Organ Surgery, Gunma University Graduate School of Medicine and maintained as described earlier^16^. NSG mice were obtained from Jackson Laboratory. Eight animals were s.c. injected with 5 × 10^6^ cells (PC14 or PC14HM cells) into the flanks of NSG mice to initiate tumors. Lung tumors were established by retro-orbital injection of 10^6^ cells. Mice were sacrificed on day 18. Lung tumor nodules were visualized by H&E staining of whole lung sections; nodules were counted in three non-adjacent sections using Eclipse E600 microscope (Nikon).

Tumor volume was determined by external measurements using equation, V = [L × W^2^] × 0.5, where V is volume, L is length, and W is width^46^.

#### *In Vivo* L-Band EPR Studies

Mice were narcotized by inhalation of air-isoflurane mixture using Ohmeda Fluotec 3 anesthetic machine and then placed into the gap of L-band (1.2 GHz) EPR spectrometer (Magnettech, Germany). The surface coil resonator was placed onto normal mammary gland or mammary tumor/tumor xenograft and spectrometer was tuned. Solutions of the pTAM probe (0.5−2 mM, 10−30 μL) in saline, pH 7.2 was injected intratissually. Immediately after injection the EPR spectra were acquired for 5−10 min.

Local anoxic conditions in place of intratissual probe injection were initiated and maintained by addition of glucose (10 mM) and glucose oxidase (100 U/ml) into injecting solution. This combination of glucose/glucose oxidase is able to consume oxygen to level less than 1 mmHg in several seconds and maintain this low value locally at least for 10 min.

### Statistical analysis

**Figure 3:**
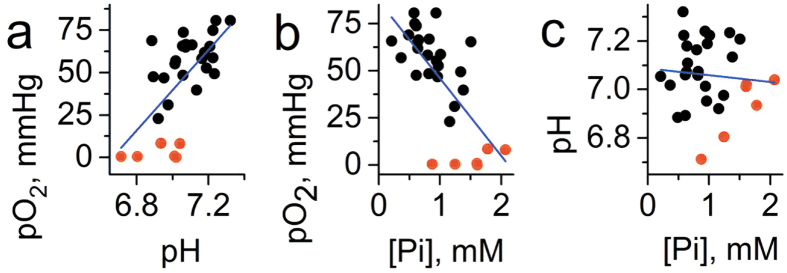
Correlation between interstitial pO_2_, pH_e_ and [Pi] values measured in normal mammary glands (n = 23) of FVB/N wild type mice. The red symbols (n = 6) correspond to the measurements performed after enzymatic oxygen depletion after i.t. injection of glucose/glucose oxidase system. (**a**) A positive correlation (r = 0.5, p = 0.014 for black symbols; r = 0.64, p = 1.8 × 10^−4^ for total data set) was found between *p*O_2_ and pH_e_. (**b**) A negative correlation was found between *p*O_2_ and [Pi] (r = −0.51, p = 0.013 for black symbols; r = −0.7, p = 2.3 × 10^−5^ for total data set). (**c**) No significant correlation was found between pH_e_ and [Pi] (r = 0.19, p = 0.4 for black symbols; r = −0.1, p = 0.65 for total data set). Blue lines represent linear fit for the total data sets.

**Figure 4:**
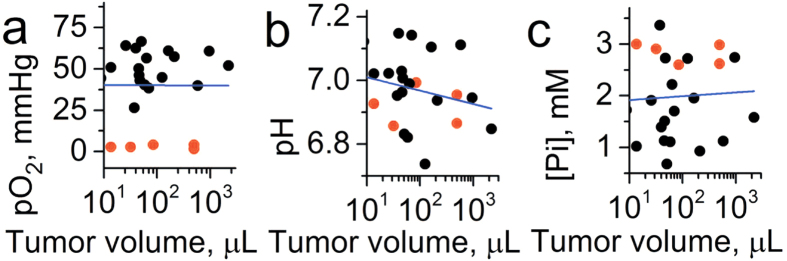
Correlation between tumor volume and interstitial pO_2_, pH_e_ and [Pi] values measured in the TME of breast cancer in MMTV-PyMT transgenic mice (n = 18). The red symbols (n = 5) correspond to the measurements performed after enzymatic oxygen depletion after i.t. injection of the glucose/glucose oxidase system. No significant correlation was found between tumor volume and (**a**) pO_2_ (r = 0.07, p = 0.76), (**b**) pH_e_ (r = −0.23, p = 0.28) and (**c**) [Pi] (r = 0.004, p = 0.99). Blue lines represent a linear fit for the total data sets.

**Figure 5:**
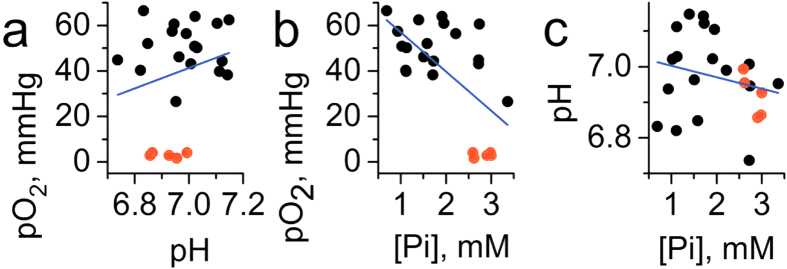
Correlation between interstitial pO_2_, pH_e_ and [Pi] values measured in the TME of breast cancer in MMTV-PyMT transgenic mice (n = 18). The red symbols (n = 5) correspond to the measurements performed after enzymatic oxygen depletion after i.t. injection of glucose/glucose oxidase system. (**a**) No significant correlation (r = 0.01, p = 0.97 for black symbols; r = 0.23, p = 0.3 for total data set) was found between pO_2_ and pH_e_. (**b**) A negative correlation was found between pO_2_ and [Pi] (r = −0.4, p = 0.079 for black symbols; r = −0.62, p = 0.001 for total data set). (**c**) No significant correlation was found between pH_e_ and [Pi] (r = −0.01, p = 0.7 for black symbols; r = −0.23, p = 0.3 for total data set). Blue lines represent linear fit for the total data sets.

**Figure 6:**
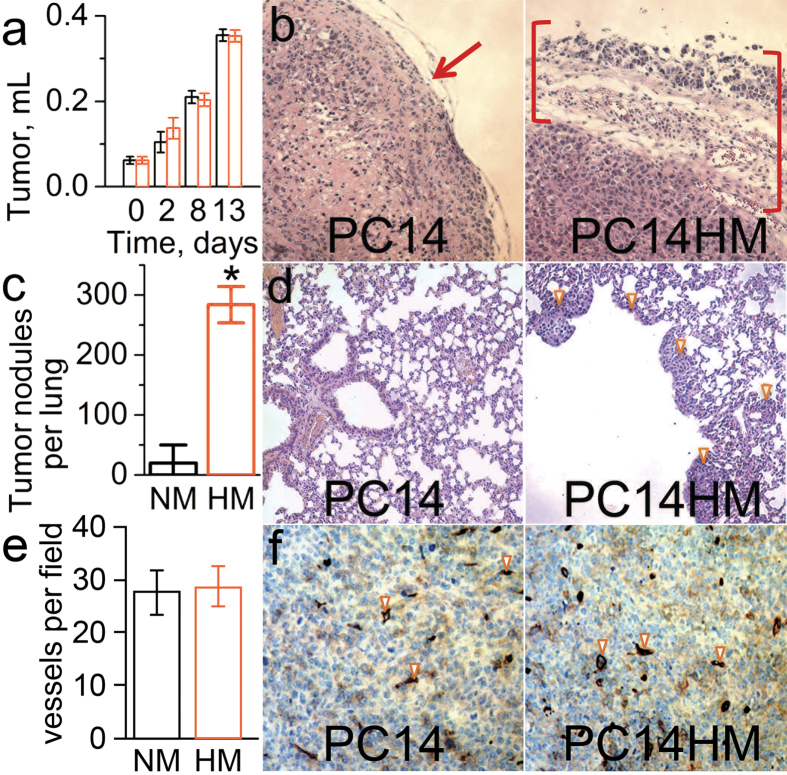
Lung metastatic properties and invasiveness of PC14 and PC14HM cells, and the growth of the corresponding sub-cutaneous tumor xenografts. (**a**) Tumor growth volume measured upon progression of non-metastatic PC14 and metastatic PC14HM tumor xenografts show no significant difference; 5 × 10^6^ PC14 or PC14HM cells were injected into flanks of NSG mice. (**b**) Photographs of the primary subcutaneous tumor tissue slides stained with H&E; appearance of PC14HM tumor demonstrates its more invasive edge (brackets) compared to the encapsulated edge of PC14 tumor (arrow). (**c,d**) NSG mice received retro-orbital injection of 10^6^ PC14 or PC14HM cells and lung metastases were enumerated 18 days later: (**c**) PC14HM cells have remarkably higher ability to form lung tumors compared to PC14 cells; n = 5. Error bars are SD. *p < 0.005; (**d**) Lung tissue sections stained with H&E. (**e,f**) CD34 immunostaining (brown) of PC14 and PC14HM tumors demonstrate no difference in vessel density: (**e**) Average number of blood vessels per field, n = 5. Error bars are SD; (**f**) Representative images of tumor sections. Arrows indicate some of CD34^+^ blood vessels. NM and HM are abbreviations for PC14 and PC14HM cells.

**Figure 7:**
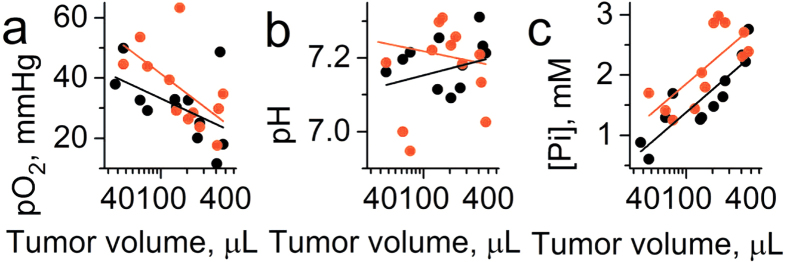
Correlation between tumor volume (TV) and interstitial pO_2_, pH_e_ and [Pi] values measured in the TME of non-metastatic PC14 (n = 12) and metastatic PC14HM (n = 12) tumor xenografts. (**a**) Significant correlation was found between pO_2_ and tumor volume for PC14HM xenografts (r = −0.59, p = 0.045 for PC14HM xenografts; r = −0.44, p = 0.15 for PC14 xenografts and r = −0.5, p = 0.014 for total set of data). (**b**) No significant correlation was found between pH_e_ and tumor volume (r = 0.02, p = 0.96 for PC14HM xenografts; r = −0.04, p = 0.9 for PC14 xenografts and r = −0.015; p = 0.94 for total set of data). (**c**) Significant correlation was found between [Pi] and tumor volume (r = 0.66, p = 0.02 for PC14HM xenografts; r = 0.9, p = 3 × 10^−5^ for PC14 xenografts and r = 0.73, p = 5 × 10^−5^ for total set of data). Note significantly higher [Pi] values for PC14HM xenografts compared to PC14 xenografts (mean [Pi] = 2.2 ± 0.2 mM and [Pi] = 1.6 ± 0.2 mM, correspondingly; p = 0.047). Black and red lines represent linear fits for PC14 and PC14HM data sets, respectively.

**Figure 8:**
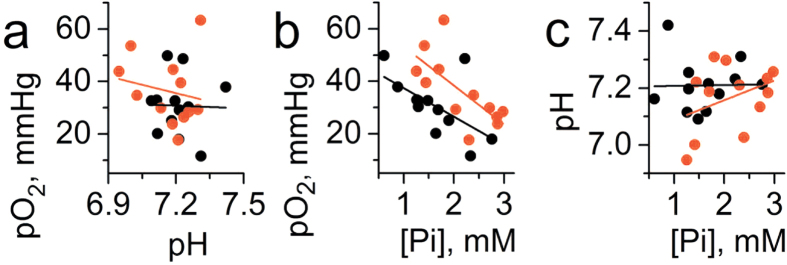
Correlation between interstitial pO_2_, pH_e_ and [Pi] values measured in the TME of non-metastatic PC14 (n = 12) and metastatic PC14HM (n = 12) tumor xenografts. (**a**) No significant correlation was found between pO_2_ and pH_e_ (r = −0.19, p = 0.56 for PC14HM xenografts; r = −0.03, p = 0.93 for PC14 xenografts and r = −0.16, p = 0.44 for total set of data). (**b**) A negative correlation was found between pO_2_ and [Pi] (r = −0.7, p = 0.01 for PC14HM xenografts; r = −0.6, p = 0.04 for PC14 xenografts and r = −0.5, p = 0.017 for total set of data). (**c**) No significant correlation was found between pH_e_ and [Pi] (r = 0.37, p = 0.23 for PC14HM xenografts; r = 0.02, p = 0.95 for PC14 xenografts and r = 0.1, p = 0.6 for total set of data). Black and red lines represent linear fits for PC 14 and PC14HM data sets, respectively.

Data processing and statistical analysis was performed using OriginPro software package. Correlation analysis of experimental data was performed using Pearson’s r Correlation test (for normally distributed datasets) and Spearman’s Rank Order Correlation (for datasets with rejected normality of data distribution). All datasets pass normality test (data are normally distributed) except total dataset of pO_2_ values for PyMT mice (see Fig. 3 black + red symbols).

## Additional Information

**How to cite this article**: Bobko, A. A. *et al*. Interstitial Inorganic Phosphate as a Tumor Microenvironment Marker for Tumor Progression. *Sci. Rep.* **7**, 41233; doi: 10.1038/srep41233 (2017).

**Publisher's note:** Springer Nature remains neutral with regard to jurisdictional claims in published maps and institutional affiliations.
